# Adiposopathy, “Sick Fat,” Ockham’s Razor, and Resolution of the Obesity Paradox

**DOI:** 10.1007/s11883-014-0409-1

**Published:** 2014-03-25

**Authors:** Harold Bays

**Affiliations:** Louisville Metabolic and Atherosclerosis Research Center, 3288 Illinois Avenue, Louisville, KY 40213 USA

**Keywords:** Adiposopathy, Adiposity, Cholesterol, Diabetes mellitus, Lipids, Metabolic syndrome, Obesity, Obesity paradox, Ockham’s razor, Sick fat

## Abstract

Among lean populations, cardiovascular disease (CVD) is rare. Among those with increased adiposity, CVD is the commonest cause of worldwide death. The “obesity paradox” describes seemingly contrary relationships between body fat and health/ill-health. Multiple obesity paradoxes exist, and include the anatomic obesity paradox, physiologic obesity paradox, demographic obesity paradox, therapeutic obesity paradox, cardiovascular event/procedure obesity paradox, and obesity treatment paradox. Adiposopathy (“sick fat”) is defined as adipocyte/adipose tissue dysfunction caused by positive caloric balance and sedentary lifestyle in genetically and environmentally susceptible individuals. Adiposopathy contributes to the commonest metabolic disorders encountered in clinical practice (high glucose levels, high blood pressure, dyslipidemia, etc.), all major CVD risk factors. Ockham's razor is a principle of parsimony which postulates that among competing theories, the hypothesis with the fewest assumptions is the one best selected. Ockham’s razor supports adiposopathy as the primary cause of most cases of adiposity-related metabolic diseases, which in turn helps resolve the obesity paradox.

## Introduction

Among rural hunter-gatherers, cardiovascular disease (CVD) is rare [1], and is independent of *nutritional quality* (i.e., amount of dietary fat) [2]. Total energy expenditure among rural hunter-gatherers does not differ from that among members of more industrialized populations [3]. Thus, reduced *nutritional quantity* and limited body fat storage best explains why hunter-gatherers have body mass index (BMI) less than 20 kg/m^2^ and minimal CVD risk factors [4], including mean total cholesterol levels of 110–150 mg/dl [5, 6].

Globally, more than one billion adults are overweight (BMI ≥ 25 kg/m^2^); at least 300 million are obese (BMI ≥ 30 kg/m^2^) [7]. Over two thirds of the US population are overweight or obese [8], with mean total cholesterol level almost twice those of hunter-gatherers (i.e., 200 mg/dl or greater) [5, 6]. When BMI increases, so does the incidence of metabolic diseases, many being major CVD risk factors [6]. Hunter-gatherer populations have low BMI, low prevalence of CVD risk factors, and a low rate of CVD. More industrialized populations have higher BMI, higher prevalence of CVD risk factors, and experience CVD as the single commonest cause of death [9]. The World Health Organization has characterized obesity and its metabolic consequences as a global epidemic [7].

This review explores the adiposopathic changes that often accompany an increased amount of body fat, and why they are best considered the “primary cause” of metabolic diseases and increased CVD risk. Understanding adiposopathy better allows clinicians to explain to patients how an increased amount of body fat leads to “sick fat” and ill-health. By recognizing the pathogenic potential of adipocytes and adipose tissue, clinicians and patients can better resolve the so-called *obesity paradox*.

## Adiposopathy and History

Approximately 400 BC, Hippocrates stated: “It is very injurious to health to take in more food than the constitution will bear, when at the same time, one uses no exercise to carry off this excess. For as aliment fills, and exercise empties the body, the result of an exact equipoise between them must be to leave the body in the same state they found it, that is, in perfect health” [10]. In the 1700 s, Morgagni published “*The seats and causes of disease investigated by anatomy*,” wherein he postulated health resulted from a vital harmony of a well-balanced function of body organs [11]. In the 1920s, obesity/central obesity was found to cluster with hyperglycemia, hypertension, hyperuricemia, and hyperlipidemia [12]. In the 1940s, Vague [13] described the association of increased abdominal obesity and the increased risk of CVD among men. Thus, at least since 400 BC, positive caloric balance has been recognized as potentially injurious to health, at least since the 1920s, central adiposity has been known to cluster with metabolic disease, and at least since the 1940s, adipose tissue distribution has been described as relevant to CVD risk.

Overall, basic medical research has consistently supported adipocyte and adipose tissue anatomic and physiologic derangements as important contributors to metabolic disease [14••]. Yet as late as the 1980s, not all were convinced that adipocytes were much more than inert cells. Adipose tissue was often perceived as a slothful organ that simply stored body fat. The relationships between an increased amount of body fat and metabolic diseases were *not* typically characterized as causal ones, but were rather characterized as “associations.” An increased amount of body fat was *not* typically characterized as a contributor to metabolic diseases; rather metabolic diseases were characterized as *comorbidities*.

The lack of definitive causal phraseology reflected the thinking that if an increase in the amount of body fat did correlate to metabolic disease and increased CVD risk, then this was either a chance finding, or was due to some inexplicable and mysterious mechanism. It was so mysterious that the clustering of increased adiposity (especially central/visceral obesity) with metabolic diseases and CVD risk factors was identified by at least 15 different syndromes, including atherothrombogenic syndrome, beer-belly syndrome, cardiovascular metabolic syndrome, chronic CVD-risk-factor-clustering syndrome, deadly quartet (obesity, hyperinsulinemia, hypertension, and dyslipidemia), disharmonious quartet, dysmetabolic syndrome, dysmetabolic syndrome X, insulin resistance syndrome, insulin resistance–dyslipidemia syndrome, metabolic cardiovascular syndrome, metabolic syndrome, metabolic syndrome X, multiple metabolic syndrome, plurimetabolic syndrome, and Reaven’s syndrome [15]. Perhaps the most cryptic moniker was the apparitional “syndrome X” [16].

In an effort to bring harmony to divergent voices, the term “metabolic syndrome” sought to define a common clustering of CVD risk factors, with increased waist circumference as the only anatomic diagnostic criterion. Yet even here, different scientific organizations had different diagnostic criteria (Table 1). In contrast to the National Cholesterol Education Program, Adult Treatment Panel III [17, 18], the International Diabetes Federation (IDF) *required* central obesity as a metabolic syndrome diagnostic criterion [19]. As such, although both the National Cholesterol Education Program, Adult Treatment Panel III and the IDF recognized central obesity as the only anatomic diagnostic criterion, the IDF more strongly identified the central role of adipose tissue in the common clustering of CVD risk factors. Additionally, the IDF included ethnicity-specific values, reflecting that different degrees of central obesity had different clinical implications, based on different genetic predispositions.

**Table 1:** Metabolic syndrome definitions

Updated National Education Cholesterol Education Program, Adult Treatment Panel III diagnostic criteria for metabolic syndrome include the presence of 3 of 5 of the following [18]: 1. Elevated waist circumference [men greater than 40 in. (102 cm); women greater than 35 in. (88 cm)] 2. Elevated level of triglycerides equal to or greater than 150 mg/dL (1.7 mmol/L) 3. Reduced high-density lipoprotein cholesterol level [men less than 40 mg/dL (1.03 mmol/L); women less than 50 mg/dL (1.29 mmol/L)] 4, Elevated blood pressure (equal to or greater than 130/85 mmHg or use of medication for hypertension) 5. Elevated fasting glucose level equal to or greater than 100 mg/dL (5.6 mmol/L) or use of medication for hyperglycemia	The International Diabetes Federation defined metabolic syndrome as the presence of central obesity with ethnicity-specific values, and any 2 of the following [19]: 1. Raised level of triglycerides greater than 150 mg/dL (1.7 mmol/L) or specific treatment for this lipid abnormality, 2. Reduced high-density lipoprotein cholesterol level less than 40 mg/dL (1.03 mmol/L) in males and less than 50 mg/dL (1.29 mmol/L) in females or specific treatment for this lipid abnormality 3. Raised blood pressure (i.e., systolic blood pressure greater than 130 mmHg or diastolic blood pressure greater than 85 mmHg or treatment of previously diagnosed hypertension 4. Raised fasting plasma glucose level greater than 100 mg/dL (5.6 mmol/L) or previously diagnosed type 2 diabetes

Although defining metabolic syndrome did bring some order, the term “metabolic syndrome” did not reflect a unified, pathophysiologic process that accounted for the clustering of metabolic disorders. Metabolic syndrome was not a “disease,” and thus was not especially conducive to cause-and-effect logistical discussions with patients. Also, because it was not a true “disease,” no therapeutic intervention and/or agent achieved regulatory approval to treat “metabolic syndrome.” Finally, the metabolic syndrome diagnosis did not appear to be a better predictor of future metabolic disease than the assessment of its individual components [20]. Given these uncertainties, in 2005, the American Diabetes Association and the European Association for the Study of Diabetes issued a joint statement questioning the clinical utility of “metabolic syndrome” [21].

Concurrently, research continued to support adipose tissue disease as relevant to a “common soil” hypothesis [6, 22]. Evidence continued to mount “confirming” that the components of metabolic syndrome were due to a single pathophysiologic process [23]. But what was this process? What was the disease? The answer was found from decades of basic science. Researchers described adipocytes and adipose tissue as metabolically active [20, 24]. Dysfunctional energy storage produced the anatomic findings of adipocyte hypertrophy/visceral fat accumulation. Adipocyte and adipose tissue endocrine/immune dysfunctions both directly, and indirectly, contributed to metabolic disease and increased CVD risk [14••].

But perhaps the best evidence was found within the sanctuarial domain of the clinician and patient. Among the commoner clinical presentations are patients who, after gaining body fat, develop increased glucose blood levels, higher blood pressure, and worsening dyslipidemia. Clinicians often intuitively deduce these metabolic diseases were caused by the increase in the amount of body fat. Hence, clinicians often recommend patients lose weight (fat) via behavior modification, nutritional interventions, and increased physical activity. Such assessments and recommendations are consistent with the scientific evidence that (1) an increase in the amount of body fat is often pathogenic, and may contribute to (“cause”) metabolic disease, and (2) patients with overweight or obesity often have metabolic disease parameters that improve with fat weight loss.

“Adiposopathy” and “sick fat” are scientific and clinical terms, respectively, that simultaneously emerged towards better aligning the scientific findings of researchers with the patient care experience of clinicians. These terms recognized the pathogenic potential of an increased amount of body fat as a primary contributor to metabolic disease and increased CVD risk. In 2004, the first peer-review publication of the term “adiposopathy” was noted in a review article of investigational antiobesity agents, wherein its conclusion stated: “An emerging concept is that the development of anti-obesity agents must not only reduce fat mass (adiposity), but must also correct fat dysfunction (adiposopathy)” [25]. This was followed by the first publication identifying “sick fat,” entitled “Adiposopathy: sick fat causes high blood sugar, high blood pressure, and dyslipidemia” [15]. Since then, consensus articles and reviews have better defined adiposopathy as it applies to clinical endocrinology [26], lipidology [27•], diabetes mellitus [28••, 29], CVD [14••], bariatric surgery [30, 31], and other inflammatory disorders [32–37].

It is now generally accepted that adipose tissue has no less pathogenic potential than other body organs, with adiposopathy being analogous to cardiomyopathy, myopathy, encephalopathy, ophthalmopathy, retinopathy, enteropathy, nephropathy, neuropathy, and dermopathy. “Cardiomyopathy” describes a “disease” wherein pathologic enlargement of heart cells and heart organ results in anatomic/functional abnormalities leading to adverse clinical consequences. Similarly, “adiposopathy” describes a “disease” wherein pathogenic enlargement of fat cells and fat organ results in anatomic/functional abnormalities leading to adverse clinical consequences. But although the pathogenic potential of adipocytes and adipose tissue has gained acceptance, such was not necessarily the case as late as 2009, when it was stated [38]:

A Type 2 diabetes mellitus patient who weighs 300 pounds (with an ideal bodyweight of 150 pounds) may develop diabetes mellitus eye disease, such as disease of the retina (a thin layer of cells lining much of the orbit). The adipose tissue in this patient likely weighs over 150 pounds, and represents this patient’s largest organ by weight. It seems unreasonable to readily accept the pathogenic potential of a tissue measured in microns (retinopathy), yet deny the pathogenic potential of an organ that can be measured in hundreds of pounds, and that may constitute over half of the patient’s bodyweight.

**Fig. 1:**
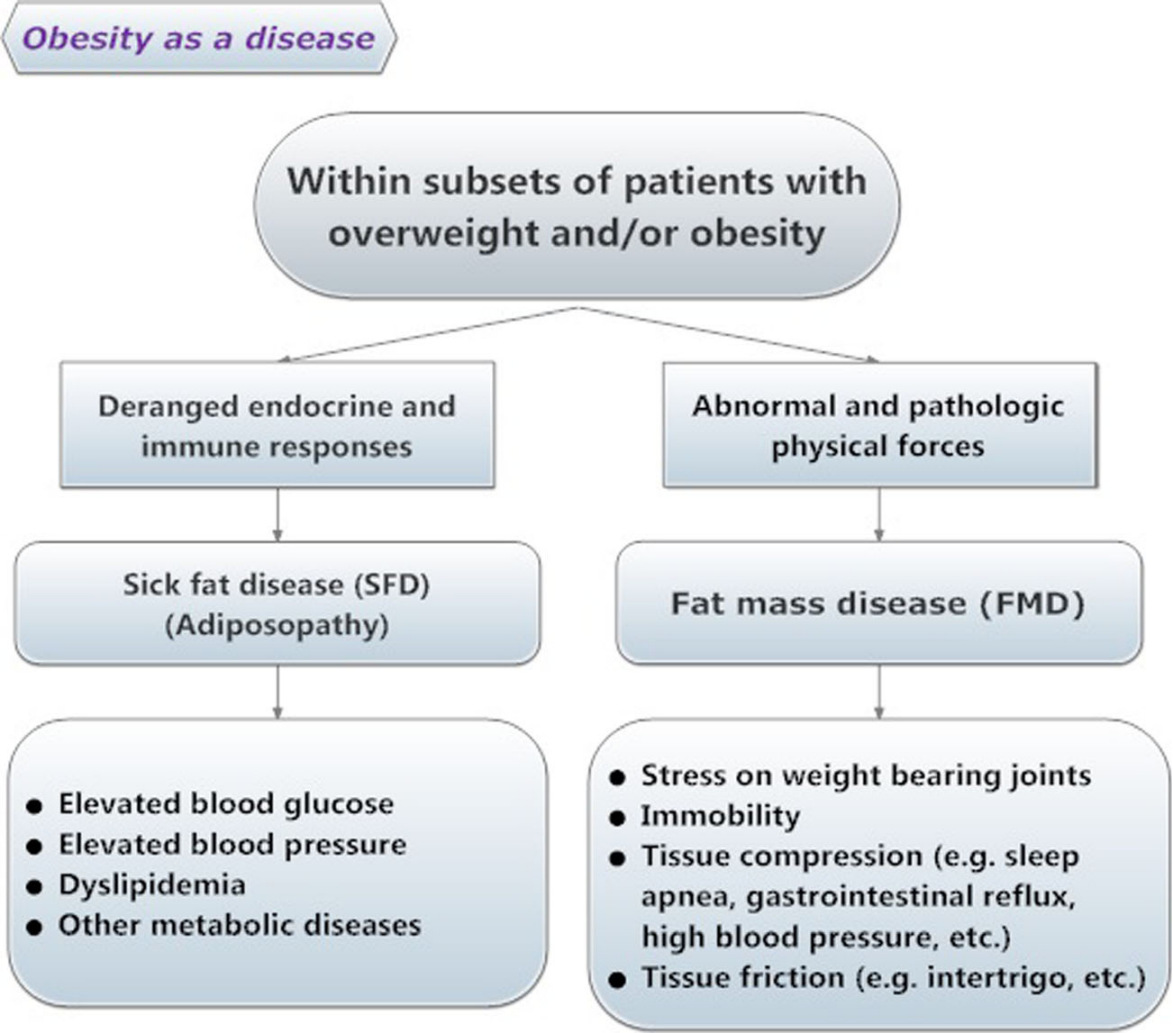
Obesity as a disease. (Copyright American Society of Bariatric Physicians 2013–2014 [41••])

Similarly, the lack of universal acceptance of adipose tissue as an organ worthy of intervention compelled authors to posit provocative manuscript titles such as: “Adipose tissue as a therapeutic target in obesity” [39]. Today, national obesity guidelines and algorithms incorporate the clinical concepts and scientific principles of adiposopathy and sick fat. The 2013 Clinical Practice Guidelines for Healthy Eating for the Prevention and Treatment of Metabolic and Endocrine Diseases in Adults notes that primary disturbances in adipose tissue anatomy and function (“adiposopathy”) are etiologic in the development of metabolic derangements [40]. A major stated focus of nutritional counseling for individuals with overweight or obesity is to correct “adiposopathy.” Gonzalez-Campoy et al. [40] recommend that nutritional counseling of individuals with overweight or obesity be aimed not only at decreasing fat mass, but also at correcting adipose tissue dysfunction (“adiposopathy”). The 2013 American Association of Bariatric Physicians Obesity Algorithm [41••] (Fig. 1) highlighted how the evaluation of patients with overweight or obesity should focus on the presence of “sick fat disease” (adiposopathy) or “fat mass disease.”

## Adiposopathy and Ockham’s Razor

The 2005 review article entitled “Adiposopathy, metabolic syndrome, quantum physics, general relativity, chaos and the theory of everything” [42] reviewed how physicists had long struggled to develop a unifying “theory of everything,” in an effort to help reconcile understandings of microsystems (via quantum physics involving molecules, atoms, and subatomic particles such as electrons and quarks) and macrosystems (through relativity theories applied to stars galaxies and the universe itself):

Thus, the challenge has been to find a simple equation that might unify the macro with the micro and bridge the gap between the classical relativity concepts and the quantum concepts of gravity/spacetime. Physicists strive towards unifying the associations between the four fundamental forces of the universe: strong forces, electromagnetic forces, weak forces and gravitational forces. This has led to superunified theories, or Theories of Everything, such as superstrings which are mathematically derived, hypothetical units that form the elementary particles of spacetime (and require the presence of multiple dimensions). In medicine, we are still grappling with the unification of the pathogenesis of the four fundamental metabolic diseases of obesity, T2DM, hypertension and dyslipidemia – and we have had our own conflicts [42].

Among the basic laws of physics applicable to individuals who are overweight or obese are the concept of chaos or uncertainty in quantum physics, which allows escape from the unyielding shackles of predetermination. Chaos allows for change via free will, which in the case of patients with overweight or obesity often includes lifestyle changes. Even the relativity formula *E* = *mc* ^2^ has clinical application:

Some obese patients believe that their metabolic fate is unavoidable, irrespective of intervention, and come to develop the defeatist belief that they will always gain fat mass, even if they reduce consumption of calories/energy. As previously described, physics dictates that in order for energy (calories) to decrease while mass continues to increase would necessitate that the speed of light decrease respective to the obese patient. This is not only impossible from a general relativity, spacetime standpoint, but is seemingly ridiculous, except to the frustrated obese patient. Thus, a major challenge to clinicians will be to dispel the notion of inevitability and instead, emphasize that free-will-based lifestyle interventions can be, and are effective [42].

Table 2 lists anatomic, pathophysiologic, and clinical manifestations of adiposopathy, manifested by disharmonious interplay of micro (cellular) and macro (organ) biological systems causing/contributing to metabolic disease and increased CVD risk. However, other contributing mechanisms potentially exist. Pathogenic infestations, via viral infections [43] or intestinal signaling from gut microbiota [44], are suggested to potentially increase the amount of body fat and cause adipose tissue dysfunction. However, little evidence supports microorganism-mediated mechanisms as superseding the fundamental importance of behavior/lifestyle-mediated positive caloric balance leading to adiposopathic endocrine and immune responses, which in turn lead to metabolic disease. Bariatric surgery’s effects on gut hormones (e.g., ghrelin) favorably treat metabolic disease, and are effects perhaps independent of fat weight loss [31]. However, although intriguing, it seems implausible to suggest that the very indication of bariatric surgery (which is to reduce the amount of body fat in patients with obesity) is the mechanism least applicable in improving metabolic diseases and reducing CVD risk [45].

**Table 2:** Adiposopathy: Causality and illustrative anatomic, pathophysiologic, and clinical manifestations. [14••, 41••]

**Causes of adiposopathy**
• Positive caloric balance
• Sedentary lifestyle
• Genetic predisposition
• Environmental causes (e.g. certain medications, viral infections, pathologic gut microbiota signaling)
**Anatomic manifestations of adiposopathy**
• Adipocyte hypertrophy
• Increased visceral, pericardial, perivascular, and other periorgan adiposity
• Growth of adipose tissue beyond its vascular supply with ischemia, cellular death, apoptosis, and inflammation
• Increased number of adipose tissue immune cells
• “Ectopic fat deposition” in other body organs (liver, muscle, pericardial fat, perivascular fat, and possibly pancreas)
**Pathophysiological manifestations of adiposopathy**
• Impaired adipogenesis
• Pathological adipocyte organelle dysfunction (e.g. “stress” to adipocyte endoplasmic reticulum, mitochondria)
• Increased circulating free fatty acids (lipotoxicity)
• Pathogenic adipose tissue endocrine responses (e.g., increased leptin, increased tumor necrosis factor-alpha, decreased adiponectin, and increased mineralocorticoids)
• Pathogenic adipose tissue immune responses (e.g., increased proinflammatory responses through increased tumor necrosis factor-alpha and decreased anti-inflammatory responses through decreased adiponectin)
• Pathogenic interactions or pathogenic cross talk with other body organs (e.g., liver, muscle, central nervous system, and vasculature.)
**Clinical manifestations of adiposopathy**
• High glucose blood levels (prediabetes, type 2 diabetes mellitus)
• Insulin resistance
• High blood pressure
• Adiposopathic dyslipidemia
○ Increased triglyceride, triglyceride rich lipoprotein, and lipoprotein remnant levels
○ Decreased high density lipoprotein cholesterol levels
○ Increased atherogenic particle number (i.e. increased apolipoprotein B)
○ Increased small dense low density lipoprotein particles
• Metabolic syndrome
• Atherosclerosis
• Fatty liver
• Hypoandrogenemia in men
• Hyperandrogenemia in women
• Polycystic ovarian syndrome, menstrual disorders, and infertility
• Hyperuricemia
• Cholelithiasis
• Glomerulopathy
• Prothrombotic state
• Cancer
• Other inflammatory diseases (e.g. worsening depression, asthma, osteoarthritis)
**Illustrative causes of metabolic diseases not due to adiposopathy**
• Type 2 diabetes mellitus may be due to hemochromatosis, chronic pancreatitis, hypercortisolism, excessive growth hormone, genetic syndromes of insulin resistance, and decreased pancreatic function (genetic syndromes, surgical excision, etc.).
• High blood pressure may be due to pheochromocytoma, primary hyperaldosteronism, hypercortisolism, hyperthyroidism, renal artery stenosis, various kidney diseases, and familial or genetic syndromes.
• Dyslipidemia may be due to untreated hypothyroidism, poorly controlled diabetes mellitus, certain types of liver or kidney diseases, and genetic dyslipidemias.

Perhaps the most compelling challenge to the central role of adipocyte and adipose tissue as being the “primary cause” of metabolic disease and increased CVD risk lies in cross talk and interactions with other body organs. The brain is the central location of behavior and hunger, and the hypothalamus is a target for weight management pharmacotherapies [28••]. It is the “flexibility” of body organs such as the liver [46] and muscle [47] to manage adiposopathic free fatty acid, endocrine, and immune onslaughts which largely determines the degree of if and when an increase in the amount of body fat ultimately contributes to metabolic diseases. So if disharmony among multiple body organs ultimately determines whether fat weight gain causes or worsens metabolic disease and CVD, then how is the “primary cause” best assigned?

In the fourteenth century, William of Ockham established Ockham’s razor: *pluralitas non est ponenda sine necessitate* (“plurality should not be assumed unnecessarily”) [48]. By emphasizing a principle of parsimony, economy, or succinctness in problem-solving, Ockham’s razor postulates that among competing theories with similar predictions, the simpler one is better. Ockham’s razor does not require evidence based on scientific results. It simply asserts that the explanation requiring the fewest assumptions is best.

Adiposopathy is the simplest explanation as to why and how increased body fat leads to metabolic disease and increased CVD risk. Scientific organizations have arguably conceded this point by designating adipose tissue as the only organ criterion (central obesity) which helps define metabolic syndrome (Table 1). The final main caveat is acknowledging not all metabolic diseases are due to adiposopathy [14••] (Table 2).

With this remaining caveat acknowledged, if Ockham’s razor is applied to clinical medicine (along with the patient-centered provision that reversibility is preferred over irreversibility when assigning causation), then a logical conclusion might be: “When multiple abnormalities promote an adverse health outcome, it is the defect most directly, simply, and reversibly associated with promoting a disease, and the defect most beneficial when corrected, which is best labeled the ‘primary cause’.” By use of this definition, among patients with overweight and/or obesity, adiposopathy is not just the primary cause, but is the commonest cause of type 2 diabetes mellitus, high blood pressure, dyslipidemia, and increased CVD risk.

Few challenge cardiomyopathy as a primary diagnosis for patients with congestive heart failure. This is even when its pathogenic potential may be dependent on genetics (hypertrophic cardiomyopathy), nutritional anomalies (alcohol, and deficiencies of thiamin, selenium, calcium, and magnesium), medications (cocaine, tricyclic antidepressants, etc.), ischemia (atherosclerosis), infections (adenovirus, staphylococcus, etc.), or toxins (cobalt). Cardiomyopathy is a primary diagnosis, even when largely promoted by diseases of other body organs diseases such as the vasculature (e.g., high blood pressure), lung (cor pulmonale), kidney (renal failure), and thyroid, and more widespread disorders such as hemochromatosis, sarcoidosis, amyloidosis, and connective tissue disease.

Similarly, adiposopathy is a primary diagnosis for patients with various metabolic diseases (Table 2) that occur or worsen with increased adiposity. This is even when its pathogenic potential may be dependent on genetics, nutritional anomalies (positive caloric balance), medications (sulfonylureas, tricyclic antidepressants, etc.), ischemia (adipocyte and adipose tissue hypoxia), viral infections (adenovirus, gut microbiota, etc.), and toxins. Adiposopathy is a primary diagnosis, even when disorders of other body organs such as the liver, muscle, and brain are operative as well.

## Adiposopathy and the Obesity Paradox

The “obesity paradox” describes seemingly contrary relationships between body fat and health/ill-health. These apparent paradoxical relationships between body fat and health/ill-health are made less paradoxical after identifying adiposopathy and sick fat as the “primary cause” of adiposity-related metabolic disease and increased CVD risk.

The *anatomic obesity paradox* suggests that abdominal adipose tissue distribution is paradoxically more pathologic than the peripheral adipose tissue distribution. If during positive caloric balance, peripheral subcutaneous adipose tissue (SAT) is able to undergo unfettered adipocyte proliferation and differentiation, then this may mitigate energy overflow to other fat depots [49]. Conversely, if SAT is not able to adequately proliferate and differentiate during positive caloric balance (a type of “acquired lipodystrophy”) [50], then the energy overflow (i.e., via increased circulating free fatty acids) may promote fat accumulation in other fat depots [e.g., visceral adipose tissue (VAT), pericardial fat, and perivascular fat]. Increased circulating free fatty acids may also infiltrate, and be “lipotoxic” to nonadipose tissue such as the liver, muscle, pancreas, heart, and kidney [51, 52]. In short, CVD may result directly from local-fat-depot-mediated adiposopathic and atherogenic processes [53–55] or indirectly through onset or worsening of metabolic diseases, many being major CVD risk factors [6, 14••, 27•, 28••, 29, 56••].

The *physiologic obesity paradox* applies when fat mass accumulation has seemingly paradoxical relationships to metabolic disease. Benign multiple symmetrical lipomatosis is manifested by increased accumulation of SAT, via increased proliferation of smaller, more functional adipocytes and increased secretion of anti-inflammatory adipokines such as adiponectin. Despite an increased amount body fat in these regions, patients with benign multiple symmetrical lipomatosis do not have an increased risk of hyperglycemia or dyslipidemia [57]. Conversely, inherited lipodystrophy is manifested by variable lack of body fat with low adiponectin levels. Because fat storage is limited, increased circulating free fatty acid levels promote lipotoxicity towards nonadipose body organs [58]. Despite having less body fat, patients with inherited lipodystrophy often have metabolic abnormalities such as hypertriglyceridemia and hyperglycemia [20, 59]. When the amount of fat is the only consideration, these genetic conditions may appear paradoxical. When considered within the context of fat function/dysfunction, then they are less paradoxical. Patients described as “metabolically healthy, but obese,” and “metabolically obese, but normal weight” [20] seem to identify physiologic obesity paradoxes. Although “metabolically healthy, but obese” patients may not actually be so “healthy” [60, 61•], they do suggest that when unfettered peripheral SAT accumulation helps mitigate adipocyte and adipose tissue pathogenic endocrine and immune responses, then this reduces the potential to cause metabolic disease [62]. Conversely, when positive caloric balance occurs in those with more limited proliferation and differentiation of peripheral SAT (“metabolically obese, but normal weight”), then energy overflow to other fat depots may promote adiposopathic endocrine and immune responses, contribute to metabolic disease, and increase CVD risk [20, 63].

The *demographic obesity paradox* includes apparent gender and ethnic paradoxes. Since the 1940s [13], women have been described to have a “paradoxical” age-adjusted reduced risk of CVD [64], compared with men, substantially because of increased proliferation and differentiation of fat cells in the less pathogenic SAT regions (“pear distribution”). This contrasts with the more limited proliferation and differentiation of fat cells in the SAT of men, resulting in more pathogenic VAT accumulation (“apple distribution”) [65]. Regarding races and ethnicities, Asians typically have fewer adipocytes, increased adipocyte size, increased amount of visceral fat, increased circulating free fatty acid levels, elevated leptin levels, increased levels of proinflammatory factors (e.g., C-reactive protein), and decreased levels of anti-inflammatory factors (e.g., adiponectin) [14••]. These classic adiposopathic findings (Table 2) help account for the onset or worsening of metabolic abnormalities and increased CVD risk among Asians, even when they are not markedly overweight. It also helps explain why compared with people of European descent, Asians have different waist circumference cutoff points for defining metabolic syndrome [19], and may require different BMI cutoff points for defining overweight/obesity [66].

The *therapeutic obesity paradox* describes the seemingly paradoxical effects of therapeutic interventions on body fat and metabolic disease. When lipoatrophic mice (with virtually no white adipose tissue) undergo transplant of functional fat, hyperglycemia, hyperinsulinemia, and muscle insulin sensitivity all improve [67]. Peroxisome-proliferator-activated receptor γ agonists increase the proliferation and differentiation of adipocytes, improve the SAT to VAT ratio, lower glucose levels, may improve lipid levels, and may reduce CVD risk [68]. These examples illustrate how adding functional fat can improve metabolic diseases that, paradoxically, are usually due to having too much body fat. Human immunodeficiency virus (HIV) patients treated with antiretroviral agents may develop loss of functional SAT relative to VAT (i.e., HIV lipodystrophy) [69]. Despite weight loss, patients “paradoxically” experience onset or worsening of metabolic disease [58]. Finally, if prognoses are based on fat function rather than fat mass, liposuction of functional SAT would not be expected to improve, and in fact does not improve, CVD risk factors such as hyperglycemia, high blood pressure, and dyslipidemia [70].

The *CVD event and*/*or intervention obesity paradox* refers to more favorable outcomes observed among patients with overweight or obesity who experience a CVD event, or undergo a cardiovascular procedure, compared with thinner individuals. This may be especially so if the comparator individuals are thin because of severe illnesses (e.g., chronic heart or lung disease, cancer), or because the thinner individuals smoke cigarettes, which would not only reduce body weight, but would also increase CVD risk [71, 72]. Men who are overweight or obese may have reduced mortality only if they are physically fit [73, 74]. Patients with chronic heart failure seem to have no mortality benefit if they have type 2 diabetes mellitus [75]. In fact, all nonsmoking type 2 diabetes mellitus patients with overweight and/or obesity may have increased mortality compared with normal-weight counterparts, with little evidence of an obesity paradox [76].

Confounders may be operative as well. An increase in the amount of body fat may heighten awareness of potential CVD risk factors when a patient presents with overweight or obesity. This may increase global medical care compared with that for those not thought to be at increased CVD risk. Additionally, many patients with overweight or obesity are treated with metabolic drug treatments (e.g., statins, antihypertensive agents, anti-thrombotic agents), which are proven to reduce CVD morbidity and mortality, and represent treatments possibly not affordable to those who are not overweight or obese [14••].

Also, although the adiposopathic mechanisms accounting for CVD in patients with overweight or obesity are more prevalent, the pathologic mechanisms accounting for CVD in thinner patients may be more pathologic. Most patients who experience a CVD event have modestly elevated cholesterol levels. Conversely, genetic dyslipidemias, such as familial hypercholesterolemia, result in profound increases in cholesterol levels, irrespective of whether the patient has an increased amount of body fat. Nonetheless, although less common, many adipose tissue independent genetic dyslipidemias are much more aggressive in promoting CVD [14••]. Similarly, the CVD of thinner patients may reflect more aggressive genetic/environmentally mediated diseases, which although less prevalent than adiposopathy, may have increased CVD morbidity and mortality.

Finally, adipose tissue is an abundant source of mesenchymal stem cells, which may provide enhanced cardiovascular autorepair [14••]. Adipose-tissue-derived stem cells have the potential to develop into adipocytes, blood vessel cells, or cardiomyocytes [14••]. After an acute CVD event or cardiovascular procedure, circulating stem/progenitor cells derived from adipose tissue may migrate to the disrupted or injured myocardial or vascular site. Individuals with overweight or obesity have greater mobilization of progenitor cells into the circulation compared with thinner individuals [77]. Thus, after an individual has experienced a CVD event or undergone a cardiovascular procedure, the greater availability of progenitor cells may afford greater cardiovascular self-repair and improved CVD outcomes [78, 79].

The *obesity treatment paradox* is the apparent lack of improved CVD outcomes with weight reduction in patients who are overweight or obese. If an increase in the amount of body fat is the “primary cause” of adiposopathic endocrine and immune responses leading to most cases of metabolic diseases and increased CVD risk, then weight reduction would be expected to improve metabolic disease and reduce CVD risk. The Look AHEAD (Action for Health in Diabetes) study was an 8-year study that compared an education-only group with an intensive therapy group in patients with overweight or obesity and type 2 diabetes mellitus [80]. Patients in the intensive therapy group lost more weight, had lower hemoglobin A_1c_ levels, and required fewer medications. However, the incidence of CVD was not reduced despite weight loss, increased exercise, and improvement in metabolic diseases [81]. This suggests intensive behavioral and lifestyle weight loss interventions are effective in treating adiposopathy and improving metabolic parameters in overweight and/or obese patients with metabolic disease. But with specific regard to reduce the risk of CVD, the best available evidence suggests that relative to behavior therapy and lifestyle changes, treatment might best be directed towards treating the adverse *consequences* of long-standing adiposopathy. This may include use of statins, antihypertensive agents, antithrombotic agents, etc. A notable exception is bariatric surgery. The degree of weight loss achieved with bariatric surgery, in addition to its other favorable hormonal/inflammatory effects, is reported to improve metabolic disease among patients who are overweight or obese, and may also decrease CVD morbidity and mortality [82]. This supports “sick fat” as a surgical disease [31].

## Conclusion

Adiposopathy is defined as pathologic adipose tissue anatomic/functional disturbances promoted by positive caloric balance in genetically and environmentally susceptible individuals which results in adverse endocrine and immune responses that both directly and indirectly contribute to metabolic disease and increased CVD risk. A clinical application of Ockham’s razor suggests adiposopathy as the “primary cause” of most cases of metabolic diseases such as high glucose levels, high blood pressure, and dyslipidemia, as well as most cases of CVD. Perhaps the most important “paradoxical” finding relative to obesity is determining when adiposopathy is best treated. Weight management interventions in patients with overweight/obesity and metabolic disease generally improve metabolic parameters. However, with the possible exception of bariatric surgery, weight management interventions (e.g., behavior and lifestyle recommendations) alone among patients with advanced metabolic disease may not reduce CVD risk. Some metabolic disease guidelines suggest the most aggressive treatments of metabolic disorders are reserved for those with the most advanced disease. For example, lipid guidelines suggest patients with CVD should receive the most aggressive lipid therapies [83–85]. However, for adiposopathy, the greatest CVD benefit of weight management via behavior and lifestyle interventions may be to prevent metabolic diseases and avoid CVD risk factors in the first place. That is because those who never develop metabolic disease and CVD risk factors would be expected to reduce their risk of CVD. Conversely, if therapy is delayed until patients with overweight and/or obesity develop metabolic diseases and CVD risk factors and experience CVD events, then this would increase their CVD risk, which at that stage, may not be as responsive to nutritional and physical activity interventions. To some, placing a higher priority on obesity prevention relative to obesity treatment may seem paradoxical. But Benjamin Franklin (1706–1790) once said: “An ounce of prevention is worth a pound of cure.” That prevention might be considered paradoxical is perhaps the greatest obesity paradox of all.
